# Analysis of arterial blood gas values when discarding different volumes of blood samples in an arterial heparin blood collector during thoracoscopic surgery

**DOI:** 10.1186/s12893-024-02501-4

**Published:** 2024-07-16

**Authors:** Ping Xue, Zhirong Sun

**Affiliations:** 1 Department of Anesthesiology, Fudan university Shanghai Cancer Center, Shanghai, China; 2 Department of Oncology, Shanghai Medical College, Fudan University, Shanghai, 200032 China

**Keywords:** Arterial blood gas, Thoracic surgery, Blood sample, Partial pressure of oxygen, Partial pressure of carbon dioxide, Total haemoglobin

## Abstract

**Background:**

Arterial blood gas analysis (ABGA) plays a vital role in emergency and intensive care, which is affected by many factors, such as different instrumentation, temperature, and testing time. However, there are still no relevant reports on the difference in discarding different blood volumes on ABGA values.

**Methods:**

We enrolled 54 patients who underwent thoracoscopic surgery and analysed differences in blood gas analysis results when different blood volumes were discarded from the front line of the arterial heparin blood collector. A paired t test was used to compare the results of the same patient with different volumes of blood discarded from the samples. The difference was corrected by Bonferroni correction.

**Results:**

Our results demonstrated that the PaO2, PaCO2, and THbc were more stable in the 4th ml (PaO2 = 231.3600 ± 68.4878 mmHg, PaCO2 = 41.9232 ± 7.4490 mmHg) and 5th ml (PaO2 = 223.7600 ± 12.9895 mmHg, PaCO2 = 42.5679 ± 7.6410 mmHg) blood sample than in the 3rd ml (PaO2 = 234.1000 ± 99.7570 mmHg, PaCO2 = 40.6179 ± 7.2040 mmHg).

**Conclusion:**

It may be more appropriate to discard the first 3 ml of blood sample in the analysis of blood gas results without wasting blood samples.

**Supplementary Information:**

The online version contains supplementary material available at 10.1186/s12893-024-02501-4.

## Introduction

Arterial blood gas analysis (ABGA) is routinely performed in emergency, intensive care, and pulmonary departments to assess acid‒base disorders or diagnose and quantify respiratory insufficiency [1]. In a clinical setting, ABGA has demonstrated very high consistency and diagnostic accuracy in acid-base disorders with experienced senior clinicians [2]. Especially for patients with traumatic lung injury, the combination of different arterial blood gas analysis variables may be an important approach for clinical decision-making [3]. Compared with routine blood and general biochemical tests, blood gas analysis has special requirements for patient preparation, specimen collection and transportation and has greater requirements for specimen quality. The results can play a direct guiding role in the diagnosis and treatment of patients by doctors. However, the neglect of some links in the specific clinical operation process results in deviation of the results, resulting in clinical misdirection, thus affecting the treatment of patients [4, 5].

Specimen quality greatly influences the accuracy of blood gas analysis results. In practice, many clinicians are sceptical of the use of ABG test results to help make sound management decisions [5, 6]. Differences in instrumentation, temperature, and testing time may affect the test results [6, 7]. For example, Sarah J et al. reported that central venous blood gas (VBG) can be used to detect and diagnose acid‒base disorders with reasonable diagnostic accuracy compared to ABGA, even in a state of shock [8]. In addition, some studies have found that a combination of algorithms based on intensive care ultrasound and ABG is beneficial in exploring the etiology of respiratory failure [9]. Different injectors often also make a difference. The accuracy and stability of ABGA differ between disposable arterial blood injectors and preheparin injectors [10]. These studies suggest that subtle differences in detection methods can have a significant impact on blood gas results and that simpler measurement methods may yield equally accurate results. Thus, to ensure the accuracy of the results, it is necessary to explore more simple, convenient and resource-saving detection methods for ABGA. The blood mixture of heparin on the front line often needs to be abandoned from the operation process [11], then ABGA can be evaluated. However, little attention has been given to the effect of discarded blood samples of different volumes on the accuracy of ABGA.

During thoracoscopic surgery, single-lung ventilation may lead to rapid changes in ABGA, which requires continuous blood gas analysis, and accurate ABGA values are important for the assessment of disease changes [12]. There are many factors affecting the results of ABGA, but the effect of discarding different volume samples in thoracoscopic surgery on the results remains unclear. To evaluate differences in the effects of discarding different volumes of blood form blood samples on the results of ABGA and minimizing blood sample resources during ABGA detection, our study analysed the results of blood gas analysis of different volumes of blood samples from 54 thoracic surgery patients.

## Methods

### Human samples

#### Inclusion criteria

Patients aged 18–80 years with one-lung ventilation during surgery in the thoracic surgery department and arterial blood samples for arterial blood gas detection were included. The above collected information can be obtained from the medical information system or nursing system.

#### Exclusion criteria

Patients with evidence of errors in sampling, processing and analysis or contraindications for blood drawing were excluded.

#### Study question

To clarify the difference on ABGA values in discarding different blood volumes in the front line.

#### Primary objectives

To compare the ABGA values in 3rd blood sample, 4th blood sample and 5th blood sample.

#### Secondary objectives

To explore the possible factors (age and sex differences) affecting the ABGA results of the 3rd, 4th and 5th ml blood samples.

56 patients participated in the evaluation of this study, and 54 patients were prospectively enrolled due to data missing in 2 patients. A total of 54 patients who underwent radial artery catheterization via thoracoscopic surgery at the Affiliated Cancer Hospital of Fudan University were enrolled from Dec 2nd, 2022, to Apr 1st, 2023. The patient underwent one-lung ventilation during surgery. The ABGA values were evaluated by the same observer.

The study involving participants were conducted in accordance with the National Institutes of Health (NIH) guidelines and were approved by Shanghai Cancer Center Institutional Review Board (SCCIRB) (2303272-8). All participants provided written informed consent before enrollment.

### Study design

**Fig. 1:**
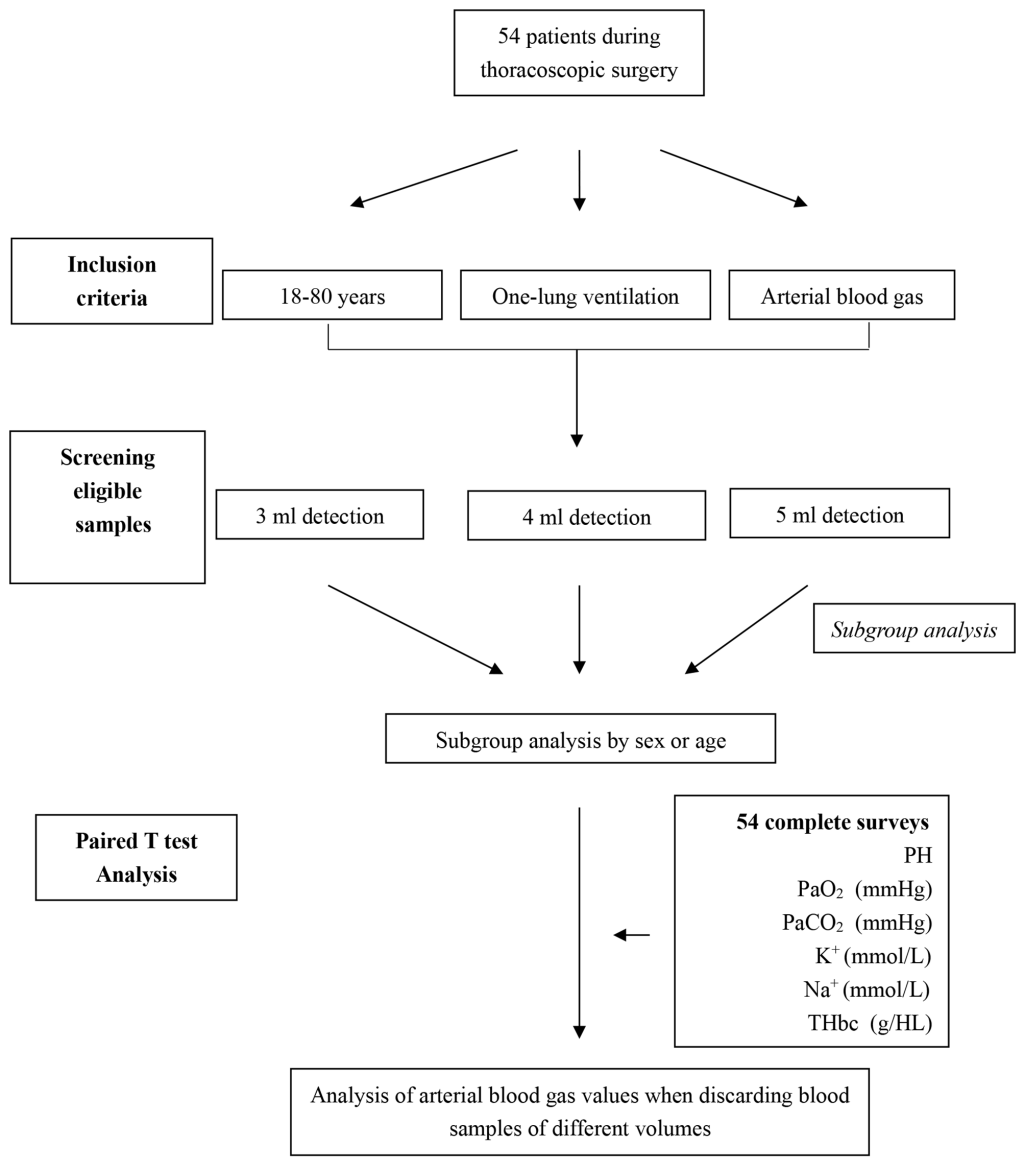
Flow chart of sampling and participant enrolment. The ABGA values (PH, PaO_2_, PaCO_2_, K^+^, Na^+^, THbc) of 54 patients during thoracoscopic surgery were test

The study design is described in Fig. 1. We registered the basic information of thoracic surgery patients who met our inclusion criteria. Blood collection was performed under the conditions of intraoperative stability, unobstructed blood collection lines, and stable ventilator mode (oxygen level 60%; PEEP 5; frequency 14). Air contact was blocked immediately after sample collection, and blood gas analysis was performed. The blood gas machine (GEM3500) was calibrated before use. Patients were ventilated with one-lung ventilation when samples were collected. To ensure that the samples were tested on the same machine, 5 ml of arterial blood was collected at one time, and the first 2 ml was discarded to exclude the influence of heparin. The 3rd ml blood sample was tested first, the 4th ml blood sample was tested immediately after the test, and the 5th ml blood sample was tested immediately after the 4th ml blood gas test. The data were collected independently by two investigators, and inconsistencies after data verification were rechecked by a third party. The extreme values found during the data analysis were further confirmed by a fourth person.

Arterial puncture: A TERUMO 20G arterial puncture needle (SR*FS2032) was used to puncture the radial artery on the nominant hand. After successful arterial puncture, the vented Medex pressure sensor was connected (MX9505T), and then the arterial blood gas was measured.

### Statistical analysis

Statistical analysis was performed using the SPSS, Inc., statistical package. Continuous variables are expressed as the mean ± standard deviation (SD). A paired t test was used to compare the results of the same patient with different volumes of blood discarded from the samples. *P* < 0.05 (marked by *) was considered significant. *P* < 0.01 (marked by **) was considered to indicate marked significance. The difference was corrected by Bonferroni correction.

## Results

### Patient characteristics

Table 1 summarizes the demographic and clinical characteristics of the patient participants. In this study, we compared the results of blood gas analysis after discarding different volumes of blood form blood samples. A total of 54 subjects were included, 55.56% of whom were male, and the average age was 59.434 ± 10.362 years. Of the 54 participants included, 40 had confirmed lung tumours (including primary and metastatic tumours), 11 had suspected lung tumours, 1 had mediastinal tumours, and 2 had thymomas **(**Table 2**)**.

**Table 1:** Characteristics of study subjects

	Subjects
Case (N)	54
Male (Male, %)	30 (55.56%)
Age (years)	59.434 ± 10.362
Primary disease (Lung tumors %)	40 (74.07%)

Note: Blood gas analysis was performed after discarding different volumes of blood samples in each participant

Abbreviation: N: number.

**Table 2:** Complication

	Subjects (*N*)
Lung tumors	40
Suspected lung tumors	11
Mediastinal tumos	1
Thymoma	2

Note: Abbreviation: N: number

### Results of discarding different volumes of blood from the arterial heparin blood collector

The blood test results are summarized in Table 3. Compared with the 3rd ml blood sample (234.1 mmHg), PaO2 concentration decreased significantly from the 4th ml (223.8875 mmHg, *p* = 0.02) to the 5th ml (221.8893 mmHg, *p* = 0.023) blood samples, and no difference was detected between the two groups of samples (*p* = 0.614). For PaCO2 or THbc, although there was a significant difference between 3rd ml (PaCO2 = 40.6179 mmHg, THbc = 10.2852 g/HL), 4th ml (PaCO2 = 41.9232 mmHg, THbc = 10.8722 g/HL) and 5th ml (PaCO2 = 40.6179 mmHg, THbc = 11.1333 g/HL) groups, the difference between the 4th and 5th ml groups was smaller. These results suggest that the blood PaO2, PaCO2, and THbc concentrations were less stable when 2 ml was discarded than when 3 ml or 4 ml was discarded.

**Table 3:** Differences of discarding different volumes blood sample in the arterial heparin blood collector

	3rd ml	4th ml	5th ml	P1	P2	P3
PH	7.2355 ± 0.0590	7.2439 ± 0.0640	7.2439 ± 0.0670	0.072	0.070	1.000
PaO2 (mmHg)	234.1000 ± 99.7570	223.8875 ± 91.1500	221.8893 ± 89.5840	0.020^*^	0.023^*^	0.614
PaCO2 (mmHg)	40.6179 ± 7.2040	41.9232 ± 7.4490	42.5679 ± 7.6410	0.001^**#^	0.000^**#^	0.037^*^
K^+^ (mmol/L)	3.3929 ± 0.4180	3.5804 ± 0.3840	3.6589 ± 0.3820	0.000^**#^	0.000^**#^	0.000^**#^
Na^+^ (mmol/L)	136.4306 ± 3.0910	136.8696 ± 2.2680	136.8893 ± 2.2730	0.137	0.099	0.882
THbc (g/HL)	10.2852 ± 1.3610	10.8722 ± 1.3180	11.1333 ± 1.2780	0.000^**#^	0.000^**#^	0.001^**#^

Note: Blood samples were collected using a 5 ml arterial heparin blood collector. We compared the results of blood gas analysis in 3nd ml, 4th ml, and 5th ml of blood samples. The blood PaO2, PaCO2, and THbc concentrations were less stable when 2 ml was discarded than when 3 ml or 4 ml was discarded. The electrolyte results may be less affected by the discarding of different volumes of blood from the samples. *P1*: 3nd ml vs. 4th ml; *P2* 3nd ml vs. 5th ml; *P3* 4th ml vs. 5th ml. *p*<0.05 (marked by *) is considered significant. *p*<0.01 (marked by **) is considered markedly significant

^#^*P*-value was survived after Bonferroni correction (*p* < 0.0167)

Abbreviation: PH: Potential of hydrogen; PaO2: Partial pressure of oxygen; PCO2: Partial Pressure of Carbon Dioxide; THbc: Total hemoglobin

We also analysed the pH and K^+^ and Na^+^ concentrations. The pH was 7.2355 in the 3rd ml group, 7.2439 in the 4th ml group, and 7.2439 in the 5th ml group. The concentrations of K^+^ were 3.3929 mmol/L in the 3rd ml group, 3.5804 mmol/L in the 4th ml group, and 3.6589 mmol/L in the 5th ml group, which were significantly greater in each group compared to the other measures. The concentration of Na^+^ was 136.4306 mmol/L in the 3rd ml group, 136.8696 mmol/L in the 4th ml group, and 136.8893 mmol/L in the 5th ml group, and differences were observed among the 3 groups. This suggests that the electrolyte results may be less affected by the discarding of different volumes of blood from the samples.

### Subgroup analysis by sex or age

We further assessed the effects of discarding different volumes of blood from the samples by conducting sex- and age-based subgroup analyses, as shown in Table 4. For the THbc analysis, similar results were observed in both groups (male vs. female), which were greater in each group compared to the other measures. There was a significant difference between the 3rd, 4th, and 5th ml blood samples in both the male and female groups, and the difference between the 4th and 5th ml blood samples was smaller. The PaO2 analysis results were similar in females but not significantly different in males. PaCO2 was significantly greater in the 4th or 5th ml blood samples than in the 3 ml blood samples, and no difference was observed between the 4th and 5th ml blood samples from males. In females, no significant difference in PaCO2 was observed among the 3 groups. However, the pH was significantly greater in the 4th or 5th ml blood samples than in the 3rd ml blood samples, and there was no significant difference between the 4th and 5th ml blood samples in females. Consistent with the overall analysis results, there were no significant differences in Na^+^ or K^+^ levels in the male and female groups.

**Table 4:** Differences of discarding different volumes blood sample in the arterial heparin blood collector subgroup analysis by sex

	3nd ml	4th ml	5th ml	P1	P2	P3
**Female (** *N* ** = 25)**						
PH	7.4028 ± 0.0620	7.4116 ± 0.0720	7.4140 ± 0.0760	0.013^*#^	0.032^*^	0.434
PaO2 (mmHg)	246.4800 ± 75.0317	231.3600 ± 68.4878	223.7600 ± 12.9895	0.006^**#^	0.004^**#^	0.247
PCO2 (mmHg)	39.2000 ± 7.7460	40.5200 ± 7.9221	40.7600 ± 8.3630	0.023^*^	0.090	0.627
K^+^ (mmol/L)	3.0960 ± 0.3529	3.2120 ± 0.2713	3.2800 ± 0.2769	0.010^*#^	0.001^**#^	0.003^**#^
Na^+^ (mmol/L)	141.7200 ± 2.9654	142.4200 ± 2.8326	142.4400 ± 2.5508	0.237	0.065	0.233
THbc (g/HL)	9.7520 ± 1.0713	10.3240 ± 1.0121	10.6480 ± 0.9747	0.000^**#^	0.000^**#^	0.004^**#^
**Male (** *N* ** = 29)**						
PH	7.3628 ± 0.0473	7.3679 ± 0.0461	7.3624 ± 0.0447	0.138	0.922	0.009^**#^
PaO2 (mmHg)	239.3448 ± 114.8114	232.6552 ± 106.6728	235.3448 ± 106.1789	0.348	0.605	0.608
PCO2 (mmHg)	44.4138 ± 5.9792	45.7931 ± 6.2984	46.8276 ± 6.0832	0.010^*#^	0.000^**#^	0.015^*#^
K^+^ (mmol/L)	3.3276 ± 0.4582	3.5034 ± 0.4314	3.5759 ± 0.4197	0.000^**#^	0.000^**#^	0.005^**#^
Na^+^ (mmol/L)	141.0000 ± 3.2950	141.4483 ± 1.7027	141.3103 ± 1.9476	0.353	0.506	0.515
THbc (g/HL)	10.7448 ± 1.4761	11.3448 ± 1.4257	11.5517 ± 1.4141	0.000^**#^	0.000^**#^	0.044^*^

Note: Blood samples were collected using a 5 ml arterial heparin blood collector. We compared the results of blood gas analysis in 3nd ml, 4th ml, and 5th ml of blood samples. A significant difference between the 3rd, 4th, and 5th ml blood samples in both the male and female groups, and the difference between the 4th and 5th ml blood samples was smaller. No significant differences in Na + or K + levels in the male and female groups. *P1*: 3nd ml vs. 4th ml; *P2* 3nd ml vs. 5th ml; *P3* 4th ml vs. 5th ml. *p*<0.05 (marked by *) is considered significant. *p*<0.01 (marked by **) is considered markedly significant

^#^*P*-value was survived after Bonferroni correction (*p* < 0.0167)

Abbreviation: PH: Potential of hydrogen; PaO2: Partial pressure of oxygen; PCO2: Partial Pressure of Carbon Dioxide; THbc: Total hemoglobin

According to the age-based subgroup analysis shown in Table 5, a more significant difference in THbc was observed in younger participants (< 50 years old). The THbc level was significantly greater in the 4th or 5th ml blood sample than in the 3rd ml blood samples, and no difference was observed between the 4th or 5th ml blood samples. Moreover, based on the age difference, there was no marked difference in the other blood gas analysis results.

**Table 5:** Differences of discarding different volumes blood sample in the arterial heparin blood collector subgroup analysis by age

	3nd ml	4th ml	5th ml	P1	P2	P3
**Age<50 (** *N* ** = 11)**						
PH	7.4036 ± 0.0516	7.4136 ± 0.0568	7.4173 ± 0.0694	0.219	0.255	0.506
PaO2 (mmHg)	244.8182 ± 123.5539	232.0909 ± 68.4878	229.2727 ± 95.2146	0.142	0.247	0.720
PCO2 (mmHg)	39.0909 ± 5.4855	39.7273 ± 8.0011	39.1818 ± 9.2176	0.490	0.947	0.548
K^+^ (mmol/L)	3.2909 ± 0.1921	3.3455 ± 0.2583	3.3727 ± 0.2533	0.311	0.260	0.695
Na^+^ (mmol/L)	140.2727 ± 4.3149	141.6364 ± 2.1574	141.2727 ± 2.1490	0.211	0.357	0.397
THbc (g/HL)	10.7091 ± 1.4775	11.4000 ± 1.5881	11.3182 ± 1.6351	0.014^*#^	0.002^**#^	0.683
**Age** **50–60 (** *N* **= 21)**						
PH	7.3848 ± 0.0685	7.3914 ± 0.0762	7.3910 ± 0.0784	0.079	0.114	0.853
PaO2 (mmHg)	255.1429 ± 90.2958	239.7143 ± 77.3603	238.5714 ± 76.9029	0.084	0.124	0.887
PCO2 (mmHg)	41.6667 ± 8.4459	42.8095 ± 7.8951	43.9524 ± 8.0962	0.079	0.009^**#^	0.023^*^
K^+^ (mmol/L)	3.2238 ± 0.4335	3.3429 ± 0.3932	3.4286 ± 0.3594	0.007^**#^	0.000^**#^	0.000^**#^
Na^+^ (mmol/L)	141.6667 ± 2.8694	142.2381 ± 2.4881	142.2381 ± 2.4679	0.253	0.208	1.000
THbc (g/HL)	10.2476 ± 1.3052	10.7667 ± 1.2338	11.0857 ± 1.2097	0.002^**#^	0.000^**#^	0.013^*#^
**Age>60 (** *N* ** = 22)**						
PH	7.3668 ± 0.0470	7.3723 ± 0.0487	7.3664 ± 0.0440	0.076	0.888	0.029^*^
PaO2 (mmHg)	229.6364 ± 93.1223	224.7273 ± 96.0487	222.1364 ± 96.2497	0.430	0.271	0.651
PCO2 (mmHg)	43.7727 ± 6.5968	45.6818 ± 6.1905	46.5000 ± 5.5869	0.001^**#^	0.000^**#^	0.068
K^+^ (mmol/L)	3.1818 ± 0.5058	3.4045 ± 0.4530	3.4818 ± 0.4697	0.001^**#^	0.000^**#^	0.000^**#^
Na^+^ (mmol/L)	141.5445 ± 2.7208	141.5000 ± 2.2414	141.7273 ± 2.2293	0.905	0.598	0.261
THbc (g/HL)	10.1091 ± 1.4342	10.7091 ± 1.3082	11.0864 ± 1.2590	0.000^**#^	0.000^**#^	0.000^**#^

Note: Blood samples were collected using a 5 ml arterial heparin blood collector. We compared the results of blood gas analysis in 3nd ml, 4th ml, and 5th ml of blood samples. Based on the age difference, there was no marked difference in the other blood gas analysis results besides “THbc”. *P1*: 3nd ml vs. 4th ml; *P2* 3nd ml vs. 5th ml; *P3* 4th ml vs. 5th ml. *p*<0.05 (marked by *) is considered significant. *p*<0.01 (marked by **) is considered markedly significant

^#^*P*-value was survived after Bonferroni correction (*p* < 0.0167)

Abbreviation: PH: Potential of hydrogen; PaO2: Partial pressure of oxygen; PCO2: Partial Pressure of Carbon Dioxide; THbc: Total hemoglobin

## Discussion

The results of blood gas analysis have important implications for the clinician’s following decisions. Our results demonstrated that the PaO2, PaCO2, and THbc results were likely to be more stable in the 4th or 5th ml blood sample than in the 3rd ml blood sample. To ensure the accuracy and stability of blood gas results, it may be more appropriate to discard the first 3 ml of blood sample in the analysis of blood gas results without wasting blood samples.

Low PaO₂ and high PaCO₂ are vital cues for critically ill patients. Previous studies have shown that a variety of factors (such as age, smoking habits, weight, etc.) may contribute to PaO2 and PaCO2 levels [13]. In addition, maternal body mass index can independently affect the fetal blood gas analysis value of scalp blood [14]. Patients with a lower BMI may have a lower PaO2, higher PaCO2, and poorer prognosis [15]. These studies suggest that individual differences in lifestyle habits or body mass index may independently influence ABGA values. Our study demonstrated that the PaO2 and PaCO2 results were more stable in the 4th or 5th ml blood samples than in the 3rd ml blood sample. However, our study did not get consistent results on the changes of PaO2 and PaCO2 levels after classifying men and women by gender. According to the sex subgroup analysis, the PaO2 concentration in females was consistent with that in the total analysis, but there was no significant difference in the PaO2 concentration in males, while the PaCO2 concentration in males was consistent with that in the total analysis. This further confirms that gender has an impact on the detection of ABGA value, which may be related to BMI, smoking habits of males and other factors.

THbc is important for the rapid evaluation of anaemia and blood transfusion indications in critically ill patients, especially for patients with active bleeding or haematological diseases [16]. Blood transfusion decisions should be made at short intervals. Previous studies have shown that many factors may affect the results of THbc, including equipment (syringe type, collection tube type, analysis equipment), analysis time, sample type and heparin dilution after sampling, but differences in blood gas detection results among different segments have rarely been reported. Our analysis of intraoperative THbc levels revealed that the THbc concentration was more stable in the 4th or 5th ml blood samples than in the 3rd ml blood sample, and the results were similar across sex and age subgroups. Therefore, when considering the wasting of blood samples, it may be more meaningful to test blood gas results after discarding 3 ml of blood.

Recently, several studies have been conducted to improve the consistency between ABGA and clinical diagnosis. For example, in patients with metabolic disorders, a method based on ultrasound imaging plus arterial blood gas analysis showed a sensitivity of 62.5% and a specificity of 98.59%, consistent with clinical diagnosis, which further improved the diagnostic accuracy of metabolic disorders [9]. The type of blood sample also has some influence on ABGA results. The majority of critically ill patients have central venous catheters, and studies have shown that the combination of VBG analysis and SpO2 provides improved bedside clinical decision-making regarding acid‒base, ventilation, and oxygenation status for critically ill patients in the emergency room and ICU compared to ABG [17, 18]. In addition, VBG is also considered to be one of the markers for assessing the adequacy of systemic oxygen delivery, and some studies suggest that ABGA and VBG comparisons can not only provide insight into the patient’s respiratory status but also assess cardiac output and the adequacy of systemic oxygen delivery [19]. Moreover, several additional methods have been evaluated to determine PaCO2; for example, the Immediate Response Mobile Analyser provided greater accuracy than did the reference measurement (ABL) for determining PaCO2 in critically ill mechanically ventilated patients [20].

Our study also has limitations. First, detection time is one of the factors affecting blood gas results, and there may be a certain amount of time error. According to the previous research, blood gas analysis of umbilical cord blood found that the PH value decreased by about 0.05 every 30 min [21]. In order to ensure the detection in the same machine, we tried to complete the blood gas detection of the 3rd, 4th and 5th blood samples in the fastest time to ensure that the time difference was as small as possible or even negligible. In addition, according to the literature, other factors (such as personal habits and BMI values) may affect blood gas analysis results [21]. Thus, more accurate conclusions may be obtained by including more factors for analysis and discussion and increasing the sample size. However, our results still have important implications for clinical practice.

Together, our study demonstrated that the PaO2, PaCO2, and THbc results were more stable in the 4th or 5th ml blood samples than in the 3rd ml blood sample during thoracoscopic surgery in patients with one-lung ventilation. To ensure the accuracy and stability of blood gas results, it may be more appropriate to discard the first 3 ml of blood sample in the analysis of blood gas results without wasting blood.

### Electronic supplementary material

Below is the link to the electronic supplementary material.

Supplementary Material 1

## Data Availability

All data generated or analyzed during this study are included in this published article [and its supplementary information files].

## References

[1] Pande RK (2021). Arterial blood gas: Bowling wide and poor wicketkeeping. Indian journal of critical care medicine: peer-reviewed. Official Publication Indian Soc Crit Care Med.

[2] Rodríguez-Villar S, Poza-Hernández P, Freigang S (2021). Automatic real-time analysis and interpretation of arterial blood gas sample for point-of-care testing: clinical validation. PLoS ONE.

[3] Carlino MV, Guarino M, Izzo A (2020). Arterial blood gas analysis utility in predicting lung injury in blunt chest trauma. Respir Physiol Neurobiol.

[4] Baird G (2013). Preanalytical considerations in blood gas analysis. Biochemia Med.

[5] Dukić L, Kopčinović LM, Dorotić A (2016). Blood gas testing and related measurements: national recommendations on behalf of the Croatian Society of Medical Biochemistry and Laboratory Medicine. Biochemia Med.

[6] Quinn LM, Hamnett N, Wilkin R (2013). Arterial blood gas analysers: accuracy in determining haemoglobin, glucose and electrolyte concentrations in critically ill adult patients. Br J Biomed Sci.

[7] Picandet V, Jeanneret S, Lavoie JP (2007). Effects of syringe type and storage temperature on results of blood gas analysis in arterial blood of horses. J Vet Intern Med.

[8] Schrauben SJ, Negoianu D, Costa C (2018). Accuracy of Acid-Base diagnoses using the central venous blood gas in the Medical Intensive Care Unit. Nephron.

[9] Panda R, Saigal S, Joshi R et al. Accuracy of critical care Ultrasonography Plus arterial blood gas analysis based Algorithm in Diagnosing Aetiology of Acute Respiratory failure. Journal of critical care medicine (Universitatea De Medicina si Farmacie din Targu-Mures). 2023;9(1):20–9.10.2478/jccm-2023-0006. 10.2478/jccm-2023-0006 PMC9987272 36890971

[10] Zhao X, Liu T, Huang M (2022). Accuracy and stability evaluation of different blood sampling methods in blood gas analysis in emergency patients: a retrospective study. J Clin Lab Anal.

[11] Hamilton RD, Crockett RJ, Alpers JH (1978). Arterial blood gas analysis: potential errors due to the addition of heparin. Anaesth Intensive Care.

[12] Ganter MT, Schneider U, Heinzelmann M (2007). How often should we perform arterial blood gas analysis during thoracoscopic surgery?. J Clin Anesth.

[13] Gillies ID, Petrie A, Morgan M (1977). Analysis of possible factors influencing PaO2 and (PaO2–PaO2) in patients awaiting operation. Br J Anaesth.

[14] Hilal Z, Mrkvicka J, Rezniczek GA (2017). Accuracy of intrapartum fetal blood gas analysis by scalp sampling: a retrospective cohort study. Medicine.

[15] Wen X, Liu P, Wu H (2014). Relation between serum myostatin with BMI and PaO₂/PaCO₂ in patients with chronic obstructive pulmonary disease. Zhong Nan Da Xue Xue bao Yi xue ban = J Cent South Univ Med Sci.

[16] Tanhehco YC (2021). Red blood cell transfusion. Clin Lab Med.

[17] Zeserson E, Goodgame B, Hess JD (2018). Correlation of venous blood gas and pulse oximetry with arterial blood gas in the undifferentiated critically ill patient. J Intensive Care Med.

[18] Chong WH, Saha BK, Medarov BI (2021). Comparing central venous blood gas to arterial blood gas and determining its utility in critically ill patients. Narrative Rev Anesth Analgesia.

[19] Sheikholeslami D, Dyson AE, Villarreal EG et al. Venous blood gases in pediatric patients: a lost art? Minerva pediatrics. 2022;74(6):789–94.10.23736/s2724-5276.21.06464-8. 10.23736/S2724-5276.21.06464-8 34530585

[20] Spelten O, Fiedler F, Schier R (2017). Transcutaneous PTCCO(2) measurement in combination with arterial blood gas analysis provides superior accuracy and reliability in ICU patients. J Clin Monit Comput.

[21] Lynn A, Beeby P (2007). Cord and placenta arterial gas analysis: the accuracy of delayed sampling. Archives Disease Child Fetal Neonatal Ed.

